# Antioxidant as an anticancer: consideration

**DOI:** 10.1186/2008-2231-20-96

**Published:** 2012-12-19

**Authors:** Viroj Wiwanitkit, Kamon Chaiyasit

**Affiliations:** 1 Visiting Professor, Hainan Medical University, Hainan, China; 2 Adjunct Professor, Joseph Ayobabalola University, Ikeji-Arakeji, Nigeria; 3 Visiting professor, Faculty of Medicine, University of Nis, Nis, Serbia; 4 Wiwanitkit House, Bangkhaae, Bangkok, Thailand; 5 Clinical Pharmacologist and Clinical Nutritionist, Food and Supplementation Medicine Society, Bangkok, Thailand

**Keywords:** Antioxidant, Anticancer, Cancer

## 

Cancer is an important non infectious disease that can be seen around the world and this medical disorder become important public health threaten. The treatment of cancer is still not fully effective and the research for improvement of cancer therapy is still required. In the present day, many new approaches for cancer treatment can be seen and the use of antioxidant in cancerous patients is an interesting topic to be discussed. In a recent editorial, the interesting question on “By what mechanism, do you expect antioxidants to kill cancer cells?” was raised [1].

Indeed, there is still no conclusive evidence for antioxidant use in cancer treatment although it might be useful in cancer prevention [2]. Therefore, the use of antioxidant in therapeutic process of cancer is a topic to be further studied. Indeed, if it is believed that the “scavenging free radical” is the main mechanism of antioxidant, the consideration should be on the pharmacodynamics of both antioxidant and standard antineoplastic drug. In case that induction of free radical is the main pharmacological process of antineoplastic drug (such as in quinine-based compound [3]), the use of antioxidant can be useless and should be avoided. However, in case that no drug interaction can be expected, the use of antioxidant can be allowed. Focusing on the specific advantage of antioxidant in cancer therapy, the antioxidant might be useful in the cancerous patients with cachexia who usually got the problems of redox dysequilibrium [4]. In this scenario, the antioxidant can help prevent Fenton reaction or reverse Warburg effect that can lead to chemotherapy resistance [5]. Hence, use of antioxidant supplementation in cancerous cachexic patient can be useful.

## Competing interest

The authors declare that they have no competing interest.

## Authors’ contributions

VW - concept, draft, final check (90%), KC - concept (10%). Both authors read and approved the final manuscript.
